# Therapeutic Potential of a Small-Molecule STAT3 Inhibitor in a Mouse Model of Colitis

**DOI:** 10.3390/cancers15112977

**Published:** 2023-05-30

**Authors:** Prema Robinson, Kelsey Montoya, Emily Magness, Emma Rodriguez, Viviana Villalobos, Nikita Engineer, Peng Yang, Uddalak Bharadwaj, Thomas Kris Eckols, David John Tweardy

**Affiliations:** 1 Department of Infectious Diseases, Infection Control & Employee Health, Division of Internal Medicine, The University of Texas MD Anderson Cancer Center, Houston, TX 77030-4009, USA; kmonto@nmsu.edu (K.M.); emily.magness@bcm.edu (E.M.); eerodriguez1@mdanderson.org (E.R.); viviana.villalobos01@utrgv.edu (V.V.); nikita@texasbehavioral.com (N.E.); ubharadwaj@mdanderson.org (U.B.); tkeckols@mdanderson.org (T.K.E.); djtweardy@mdanderson.org (D.J.T.); 2 Department of Clinical Cancer Prevention, The University of Texas MD Anderson Cancer Center, Houston, TX 77030-4009, USA; yang.x.peng@gsk.com; 3 Department of Molecular & Cellular Oncology, The University of Texas MD Anderson Cancer Center, Houston, TX 77030-4009, USA

**Keywords:** STAT3, inflammatory bowel disease, ulcerative colitis, small molecule targeting

## Abstract

**Simple Summary:**

We used the dextran sodium sulfate (DSS) murine model of colitis, which is widely used in preclinical studies, to determine the contribution of STAT3 to IBD. STAT3 has two isoforms: (STAT3α; which has pro-inflammatory and anti-apoptotic functions, and STAT3β; which attenuates the effects of STAT3α). In the current study, we determined the contribution of STAT3 to IBD across all tissues by examining DSS-induced colitis in mice that express only STAT3α and in mice treated with TTI-101, a direct small-molecule inhibitor of both isoforms of STAT3. We demonstrated that DSS-induced colitis is more severe in mice that express only STAT3α compared to wild-type mice and that administration of TTI-101 completely prevented DSS-induced colitis as well as reversed upregulation of CRC-associated genes.

**Abstract:**

Background and Aims: Inflammatory bowel disease (IBD) predisposes to colorectal cancer (CRC). In the current studies, we used the dextran sodium sulfate (DSS) murine model of colitis, which is widely used in preclinical studies, to determine the contribution of STAT3 to IBD. STAT3 has two isoforms: (STAT3 α; which has pro-inflammatory and anti-apoptotic functions, and STAT3β; which attenuates the effects of STAT3α). In the current study, we determined the contribution of STAT3 to IBD across all tissues by examining DSS-induced colitis in mice that express only STAT3α and in mice treated with TTI-101, a direct small-molecule inhibitor of both isoforms of STAT3. Methods: We examined mortality, weight loss, rectal bleeding, diarrhea, colon shortening, apoptosis of colonic CD4+ T-cells, and colon infiltration with IL-17-producing cells following 7-day administration of DSS (5%) to transgenic STAT3α knock-in (STAT3β-deficient; ΔβΔβ) mice and wild-type (WT) littermate cage control mice. We also examined the effect of TTI-101 on these endpoints in DSS-induced colitis in WT mice. Results: Each of the clinical manifestations of DSS-induced colitis examined was exacerbated in ΔβΔβ transgenic versus cage-control WT mice. Importantly, TTI-101 treatment of DSS-administered WT mice led to complete attenuation of each of the clinical manifestations and also led to increased apoptosis of colonic CD4+ T cells, reduced colon infiltration with IL-17-producing cells, and down-modulation of colon mRNA levels of STAT3-upregulated genes involved in inflammation, apoptosis resistance, and colorectal cancer metastases. Conclusions: Thus, small-molecule targeting of STAT3 may be of benefit in treating IBD and preventing IBD-associated colorectal cancer.

## 1. Introduction

Inflammatory bowel disease (IBD) causes chronic and debilitating gastrointestinal morbidity and can be life-threatening [1,2,3]. IBD also leads to a risk of colorectal cancer (CRC) that is up to 30-fold higher than that of the general population [4,5]. In addition, CRC in patients with IBD is more advanced at diagnosis and has a higher mortality rate compared to CRC in patients without IBD [6].

The etiology of IBD has not been established; however, several genes, including STAT3 have been demonstrated as risk factors in genome-wide association studies [7]. STAT3 in three cell lineages—myeloid cells, enterocytes, and T cells—has been found to contribute to colitis in mice and humans [8,9,10,11,12,13,14], but in contrasting ways. Chronic colitis due to increased Th1 cells and decreased IL-10 responses is known to result in mice that have STAT3 genetically deleted in myeloid cells (neutrophils and macrophages) [15]. Futhermore enhanced susceptibility to experimental colitis has also known to occur in mice that have STAT3 genetically deleted in enterocytes [15]. The above studies indicate that STAT3 in myeloid cells and enterocytes protects against colitis. In contrast, STAT3 activation in CD4+ T cells leads to the prevention of apoptosis of pathogenic CD4+ T cells with the ensuing manifestation of chronic intestinal inflammation [15,16,17], suggesting that STAT3 activation in CD4+ T cells promotes colitis. Uncertainty remains, however, regarding which cellular compartment(s) STAT3 is most critical for IBD development and whether STAT3 in aggregate across all cells promotes or prevents IBD. The two isoforms of STAT3; α (p92) and β (p83), are expressed in most cells in a 4:1 ratio (α:β) [18,19]. The distinct contributions of each isoform, as well as STAT3 itself, to embryonic development, inflammation, and apoptosis [20,21,22] have been demonstrated in mice that are deficient in one or both isoforms [20,22]. In particular, STAT3α is pro-inflammatory and has anti-apoptotic effects, while STAT3β attenuates STAT3α’s pro-inflammatory and anti-apoptotic effects. In this study, we induced colitis using DSS, a heparin-like polysaccharide dissolved and administered in drinking water. The DSS-induced colitis model is widely used to model IBD in mice [23] and is known to induce colonic epithelial damage, thereby mimicking some of the features of IBD. We examined the contribution of STAT3 across all cell compartments in the dextran sodium sulfate (DSS) model of colitis [23] by modulating levels of STAT3 activity both genetically and pharmacologically. We demonstrated that DSS-induced colitis is more severe in mice that express only STAT3α compared to wild-type mice and that administration of a selective, small-molecule inhibitor of STAT3, TTI-101, completely prevented DSS-induced colitis as well as reversed upregulation of CRC-associated genes. These studies indicate that small-molecule targeting of STAT3 may be of benefit in the treatment of colitis and suggest its potential for use in patients refractory to current therapies as well as in the prevention of colitis-associated colorectal cancer.

## 2. Materials and Methods

### 2.1. DSS-Induced Colitis Models

Experimental colitis was induced by administration of 5% DSS in drinking water for 7 days to 8-week-old STAT3α knock-in/STAT3β-deficient (ΔβΔβ) mice (kind courtesy of Dr. Valerie Poli, Department of Genetics, Biology, and Biochemistry, University of Turin, Italy [24]) and cage-control, wild-type (WT) mice (both BALB/c background) as described previously [25]. Controls consisted of ΔβΔβ and cage control WT mice (both BALB/c background) administered plain drinking water. In the second set of experiments; 8-week-old WT BALB/c mice procured from Jackson Laboratories (Bar Harbor, ME, USA) were administered 5% DSS in drinking water for 7 days and treated with TTI-101 (100 mg/kg body weight in DMSO (100 μL), intraperitoneally (IP) daily for 7 days, or without TTI-101 [DMSO (100 μL) IP daily for 7 days]. We used TTI-101 at 100 mg/kg IP, as this was determined in previous studies to be the maximum tolerated dose [26]. Controls consisted of mice administered plain drinking water with and without TTI-101 treatment. TTI-101 is a small-molecule inhibitor of STAT3 provided by Tvardi Therapeutics, Inc. and used as described previously [25,26,27,28,29,30,31,32,33,34,35,36]. In both sets of experiments, rectal bleeding, and diarrhea were scored on a scale of 0 to 3. On day 7, mice were euthanized by an overdose of anesthesia followed by cervical dislocation; colons were harvested and the lengths were measured. Experiments examining DSS-induced colitis in ΔβΔβ vs. WT mice and in TTI-101-treated vs. vehicle-treated mice were each performed twice. Flash-frozen colons were used for RNA isolation and microarray analyses and for measurement of p-Y STAT3 by the Luminex assay, while 10% formalin-fixed, paraffin-embedded colons were sectioned, stained with hematoxylin and eosin (H&E), and used to determine the inflammation score. Inflammation was graded on the H&E-stained sections on a scale of 0–3 as follows: 0 = no inflammatory cells detected in the section; inflammatory cells comprising 1 = <10% of the section; 2 = inflammatory cells comprising 10–25% of the section; and 3 = inflammatory cells comprising > 25% of the section. The formalin-fixed paraffin-embedded colon sections were also used for IHC and TUNEL analyses, as detailed below.

### 2.2. Immunohistochemical and TUNEL Analysis

Immunohistochemistry (IHC): Briefly, formalin-fixed paraffin-embedded colon sections were deparaffinized using three 10-min incubations with xylene with periodic shaking, followed by one 10-min incubation each with 100%, 90%, and 80%, and 70% ethanol. The slides were then washed twice with 1XPBS (5 min each wash) and then treated with 3.0% hydrogen peroxide (prepared in water) for 5 min at room temperature in order to quench peroxidase. The slides were then immediately washed twice (10 min each wash) with 1XPBS and then incubated with polyclonal rabbit antibodies against CD4 or IL-17A (cat #BS 0647-R and BS 1183-R, Bioss, Woburn, MA, USA) at dilutions of 1/250 and 1/1000, respectively, and developed using the automated bond polymer refine detection kit (cat #DS 9800, Leica Biosystems, Buffalo Grove, IL, USA). Adjacent sections stained with polyclonal control rabbit antibodies were included as controls.

### 2.3. Tunel Assay

In order to determine apoptotic cells, the TUNEL assay was performed using the ApopTag Plus Peroxidase In Situ Apoptosis Detection Kit (EMD Millipore, Billerica, MA, USA). according to the manufacturer’s instructions. Briefly, formalin-fixed paraffin-embedded colon sections were deparaffinized and then treated with 3.0% hydrogen peroxide as above. The slides were then treated with equilibration buffer for 1 min, followed by draining of the equilibration buffer and treatment of each section with terminal deoxynucleotidyl transferase enzyme for 1 h at 37 °C. The enzyme treatment was stopped by treatment with stop-wash buffer, followed by washing three times with 1XPBS (1 min per wash). The sections were then treated with antidigoxigenin conjugate for 30 min, followed by 1 min of washing with 1XPBS and treatment with peroxidase substrate for 3–5 min. The sections were washed three times with distilled water (1 min per wash), counterstained with hematoxylin, mounted, and viewed under a light microscope. in order to determine the percentage of CD4+ cells undergoing apoptosis. Five randomly chosen high-power fields (400×) were used to count 500–1000 CD4+ stained cells that were TUNEL positive. The data are presented as a percentage of positive cells ± SEM for each group.

### 2.4. Luminex Assay and IHC for Measurement of Y705-Phosphorylated (p)-STAT3 and Total-STAT3

Millipore (Milliplex) Luminex bead-based assay kits were used to determine levels of pY-STAT3 and total-STAT3 on portions of snap-frozen colons. Protein levels of total STAT3 and pY-STAT3 were normalized to total protein levels quantitated using the Bradford method (cat #500-0006, Bio-Rad, Hercules, CA, USA). For pY-STAT3 IHC, sections of FFPE colon blocks were de-waxed, subjected to antigen recapture, and incubated with a primary rabbit monoclonal antibody against pY-STAT3 [Cat no. Phospho-Stat3 (Tyr705) (D3A7) XP^®^ Rabbit mAb #9145, Cell Signaling Technology, Beverly, MA, USA; dilution of 1/200 was used]. Slides were evaluated by light microscopy for the percent of pY-STAT3 staining nuclei separately within epithelial cells and within surrounding stromal/inflammatory cells in a blinded fashion.

### 2.5. RNA Isolation and mRNA Expression Analysis

Total RNA was isolated from portions of the snap-frozen colon by homogenization in QIAzol lysis reagent (Qiagen, Hilden, Germany) using a Tissue Lyser LT (Cat No./ID: 69980, Qiagen) to disrupt the tissue. The quality and quantity of the RNA were assessed by gel electrophoresis and a NanoDrop spectrophotometer (Thermo Fisher Scientific, Waltham, MA, USA). Synthesis, labeling, and hybridization of cDNA were performed on the Affymetrix platform at the MD Anderson Sequencing and Microarray Facility with RNA from 3–5 mice from each group following Affymetrix’s recommended protocols and the GeneChip mouse exon 1.0 ST array (Affymetrix, Santa Clara, CA, USA). The software used was Transcriptome Analysis Console 4.0, which normalizes and applies the log2 function to array signals using a robust multiarray averaging (RMA) algorithm. Differentially expressed transcripts between different groups were determined by a fold change in absolute value equal to or greater than 1.1 and a *p*-value obtained from the eBayes analysis less than 0.05. To support the visual exploration of data, a heatmap was generated in R (version 3.4.1; http:///www.r-project.org/; accessed 12 April 2018. The array data has been uploaded onto the NCBI GEO database with accession number GSE117355.

### 2.6. Statistical Analyses

Unless indicated otherwise, the data presented are the mean ± SEM for each group. Statistical analyses were performed with GraphPad Prism 7.03. The statistical differences between groups were determined using the Mann-Whitney test or Kruska-Wallis test when the data determined by the Kolmogorov-Smirnov test or the Shapiro-Wilk test did not follow a normal distribution. All other analyses were performed using the one-way ANOVA, followed by a pairwise comparison using Tukey’s post-hoc test. Survival data was analyzed using Kaplan-Meier analysis. Significance was set at *p* ≤ 0.05.

## 3. Results

### 3.1. Effect of STAT3β Deletion on DSS-Induced Colitis in Mice

**Figure 1:**
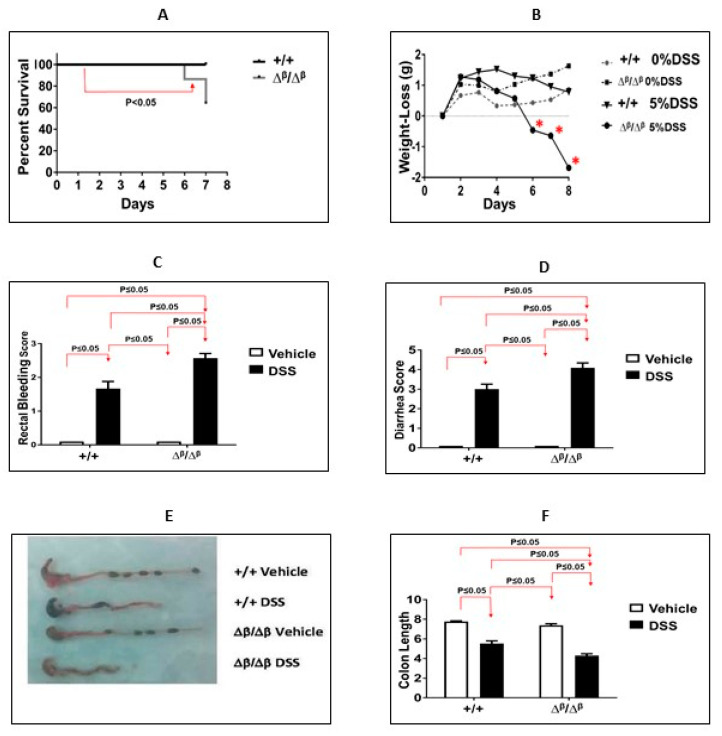
Mortality, weight loss, diarrhea, and rectal bleeding are increased, and colon length is decreased in ΔβΔβ comparison to cage-control +/+ mice (both BALB/c background). (**A**) Survival in the DSS ΔβΔβ mice group vs. the DSS +/+ mice group (*p* ≤ 0.05, Kaplan-Meier analysis, n = 9–22). (**B**) Weight loss in the DSS ΔβΔβ mice compared to DSS +/+ (data expressed as the mean ± SD; *, days 5, 6, and 7 (all *p* ≤ 0.05; ANOVA followed by Tukey’s post-hoc test; n = 9–22). (**C**) Rectal bleeding; (**D**) diarrheal score; and (**E**,**F**) colon length were assessed on day 7. Results are expressed as the mean ± SEM of two separate experiments ((**C**,**D**) *p* ≤ 0.05; Mann Whitney Test; (**F**) ANOVA followed by pair-wise comparison with Tukey’s test ((**C**,**D**,**F**); all n = 6–13)).

STAT3α is pro-inflammatory and has anti-apoptotic effects, while STAT3β attenuates STAT3α’s pro-inflammatory and anti-apoptotic effects. We determined the pathogenesis of DSS-colitis in transgenic STAT3α knock-in/STAT3β-deficient (Δ^β^Δ^β^) mice which have STAT3β deleted and hence have resultant unopposed pro-inflammatory and anti-apoptotic actions of STAT3α in all tissues. We hence determined the severity of DSS-colitis in the transgenic versus wild-type littermate cage control (+/+) mice. We administered DSS (5%) in their drinking water for 7 days and then examined various manifestations such as mortality, weight loss, rectal bleeding, diarrhea, colon shortening, and apoptosis of CD4+ T-cells (Figure 1). We determined that the severity of DSS-colitis in the transgenic ΔβΔβ mice was more exacerbated compared to wild-type littermate cage control (+/+) mice; (A) mortality in DSS, ΔβΔβ mice vs. DSS +/+ mice (36% vs. 0%; Figure 1A; *p* ≤ 0.05, Kaplan-Meier analysis; n = 9–22), (B) weight loss in DSS, ΔβΔβ mice (detected on days 5, 6, and 7; Figure 1B; *p* ≤ 0.05, ANOVA followed by pair-wise comparison with Tukey’s test), while +/+ mice did not manifest with weight loss (*p* > 0.05, ANOVA followed by pair-wise comparison with Tukey’s test, n = 9–22), (C) rectal-bleeding scores in DSS, ΔβΔβ mice was significantly higher vs. DSS +/+ mice (2.57 ± 0.14 vs. 1.67 ± 0.21; Figure 1C; *p* ≤ 0.05, Mann Whitney Test, n = 6–13), (D) diarrheal-scores in DSS, ΔβΔβ mice, also was significantly higher vs. DSS +/+ mice (4.0 ± 0.25 vs. 3 ± 0.25; Figure 1D; *p* ≤ 0.05, Mann Whitney Test, n = 6–11). These more severe clinical manifestations of DSS-induced colitis in ΔβΔβ mice were accompanied by a more pronounced shortening of their colons, which were 22% shorter in DSS ΔβΔβ mice compared to DSS +/+ mice (4.3 ± 0.19 cm vs. 5.54 ± 0.25 cm; Figure 1E,F; *p* ≤ 0.05, ANOVA followed by pair-wise comparison with Tukey’s test, n = 6–13).

**Figure 2:**
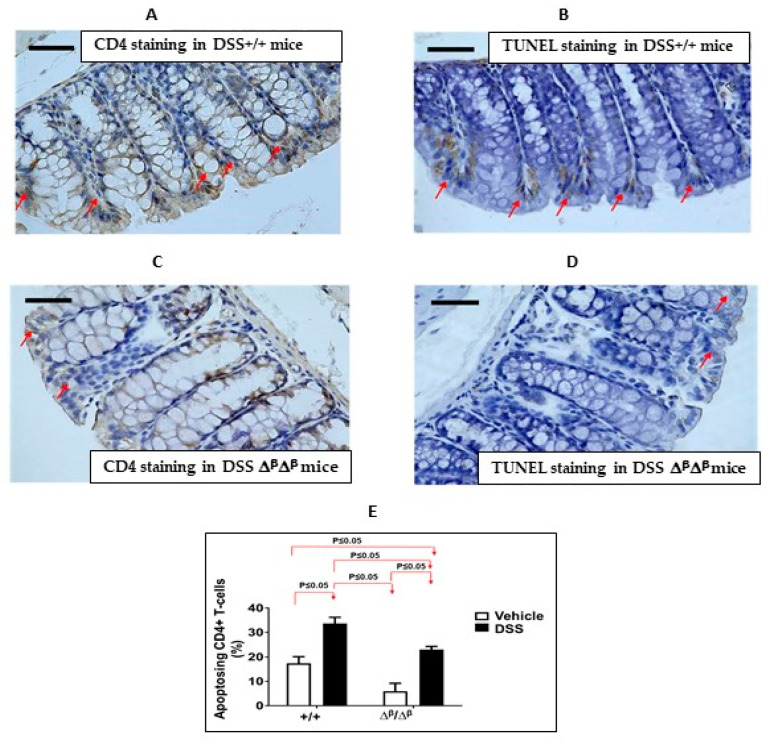
Apoptosis of CD4+ cells is decreased in the colons of ΔβΔβ mice compared to cage-control +/+mice (both BALB/c backgrounds). (**A**,**B**) Representative photomicrographs of CD4− (**A**) and TUNEL-stained sections (**B**) of colon tissue obtained from DSS+/+ and (**C**,**D**) Representative photomicrographs of CD4− (**C**) and TUNEL-stained sections (**D**) of colon tissue obtained from DSS ΔβΔβ mice (magnification 400×; scale bar represents 50 microns). Red arrows depict cells positively stained for CD4 (cell surface staining) or TUNEL (nuclear staining). (**E**) The percentage of TUNEL-positive CD4+ cells expressed as % of apoptotic CD4 T-cells ± SEM. The percentage of apoptotic CD4 T-cells was determined in ≥5 fields from n = 3–4 mice (*p* ≤ 0.05, Mann-Whitney test).

While apoptosis in each of the three cell lineages shown to contribute to DSS-induced colitis—myeloid cells, enterocytes, and CD4+ T cells—might be expected to be reduced in ΔβΔβ mice vs. +/+ mice, reduced apoptosis only within pathogenic CD4+ T cells would be consistent with worsening of DSS-induced colitis as we observed in ΔβΔβ mice. To determine if this was the case, we assessed the percentage of apoptosing CD4+ cells (Figure 2A–D). The percent of apoptosing CD4+ cells was significantly decreased (by 38%) in the colons of DSS ΔβΔβ mice vs. DSS +/+ mice (23.1% ± 1.3% vs. 33.7% ± 2.5%; Figure 2E; *p* ≤ 0.05, Mann-Whitney test, n = 3–4). These results implicate that the exacerbation of DSS-induced colitis in ΔβΔβ mice may be due, in part, to decreased apoptosis/improved survival of CD4+ T cells. This further suggests that the contribution of unopposed STAT3α to DSS-induced colitis in CD4+ cells supersedes its contribution in myeloid and/or epithelial cells.

### 3.2. Effects of TTI-101 Treatment on DSS-Induced Colitis

**Figure 3:**
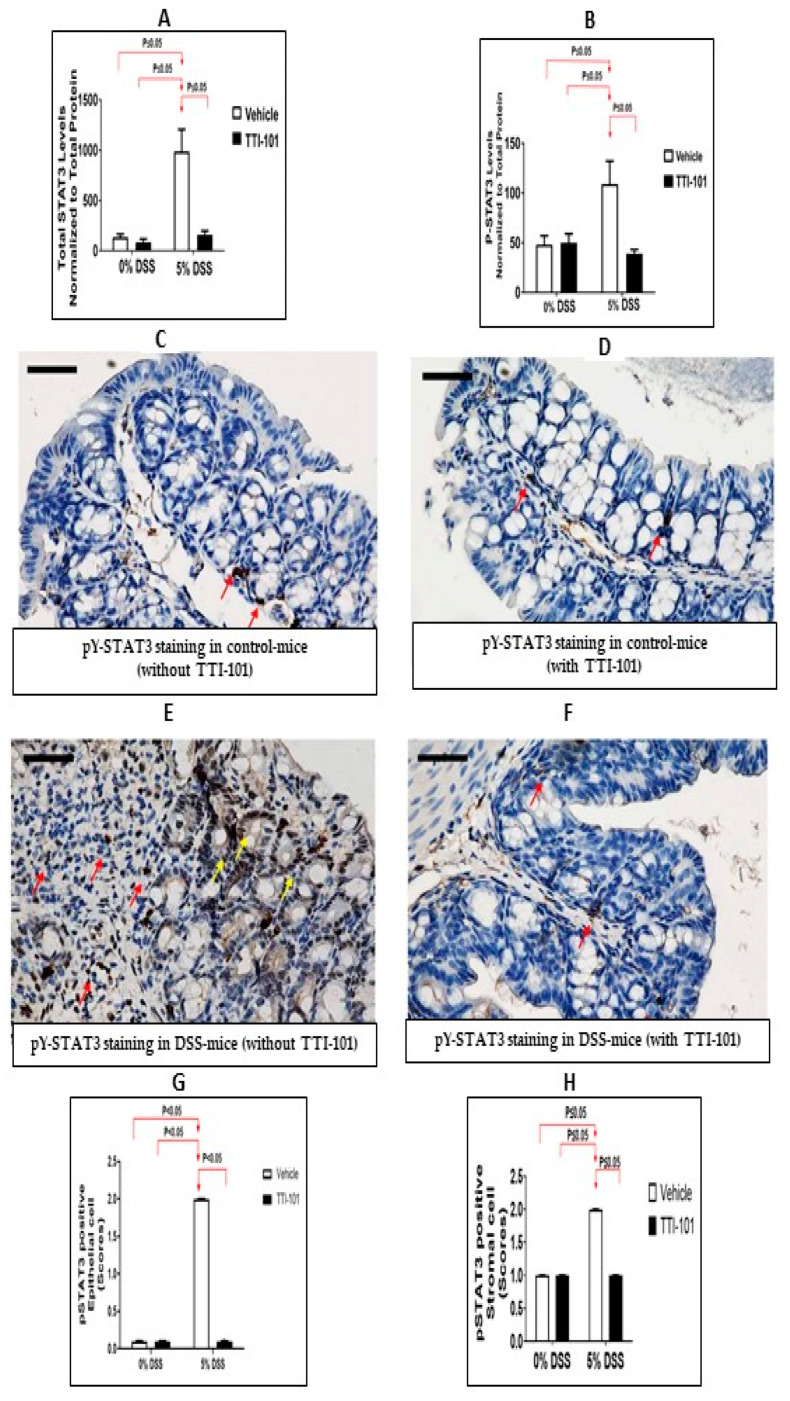
TTI-101 treatment decreases levels of total STAT3 and pY-STAT3 proteins in mouse colons. (**A**) Total STAT3 and (**B**) pY-STAT3 protein levels were measured by Luminex assay in the colons of mice that received drinking water with no DSS (0%) or 5% DSS with and without TTI-101. Both total STAT3 and pY-STAT3 levels are normalized to total protein (**A**,**B**), and the means ± SD of the ratios are plotted (*p* ≤ 0.05 for both, ANOVA followed by Tukey’s post-hoc test; n = 3–6). Representative photomicrographs (magnification 400×) of pY-STAT3-stained sections of colons from (**C**) mice that received plain water with no TTI-101 (vehicle (DMSO)), (**D**) mice that received plain water plus TTI-101, (**E**) DSS mice with no TTI-101 (vehicle (DMSO)), or (**F**) DSS mice plus TTI-101 Red arrows depict cells positively stained for pY-STAT3 (nuclear localization) within the stroma, and yellow arrows depict cells positively stained for pY-STAT3 within the epithelium. The scale bar represents 50 microns. (**G**,**H**) percentages of pY-STAT3-positive nuclei in epithelial cells (**G**) and stromal cells (**H**) in the colons of mice that received plain water or water with DSS and were treated with vehicle (DMSO) or TTI-101. Results are expressed as the mean ± SEM. The percentage of pY-STAT3-positive nuclei was determined in ≥5 fields from n = 2–5 mice (*p* ≤ 0.05 for both, Kruska-Wallis test).

We next studied the contribution of STAT3 to DSS-colitis by pharmacological modulation using a small-molecule inhibitor of STAT3 identified by our group and being developed by Tvardi Therapeutic, Inc. TTI-101 targets the Src-homology (SH) 2 domain of STAT3 [26,36,37] and was shown to target STAT3 in mouse models of inflammation, fibrosis and cancer [26,27,28,29,30,31,32,33,34,35,36]. We previously performed extensive pre-clinical evaluations of TTI-101 looking for off-target and on-target adverse effects, which revealed a high degree of specificity of TTI-101 for STAT3 [26,27]. In addition, pharmacotoxicology studies in rats, dogs, and monkeys revealed no clinical or organ-specific toxicity, which enabled its use in Phase I studies in cancer patients and patients with idiopathic pulmonary fibrosis that have advanced to multiple Phase II studies. Using Luminex assays, we confirmed a previous report [11] that levels of total STAT3 and pY-STAT3 were increased in the colons of mice administered DSS (Figure 3A; *p* ≤ 0.05, ANOVA followed by Tukey’s post-hoc test, n = 3–6) and (Figure 3B; *p* ≤ 0.05, ANOVA followed by Tukey’s post-hoc test; n = 3–6). Treatment with TTI-101 reduced total STAT3 by 81% and pY-STAT3 by 64% compared to that in control-treated DSS mice, thereby normalizing the levels of both (Figure 3A,B; *p* ≤ 0.05 for both, ANOVA followed by Tukey’s post-hoc test, n = 3–6). To confirm and extend these results, we performed pY-STAT3 IHC staining of colon sections (Figure 3C–H). Colons from mice that received DSS-containing water demonstrated increased numbers of epithelial and stromal cells with pY-STAT3-positive nuclei cells compared to colons from mice given plain water (*p* ≤ 0.05 for both, Kruska-Wallis test; n = 2–5). TTI-101 treatment reduced pY-STAT3-positive nuclei in mice given DSS-containing water in epithelial cells (*p* ≤ 0.05, Kruska Wallis test; n = 2–5) and in stromal cells (*p* ≤ 0.05, Kruska Wallis test; n = 2–5) to levels identical to mice given plain water.

**Figure 4:**
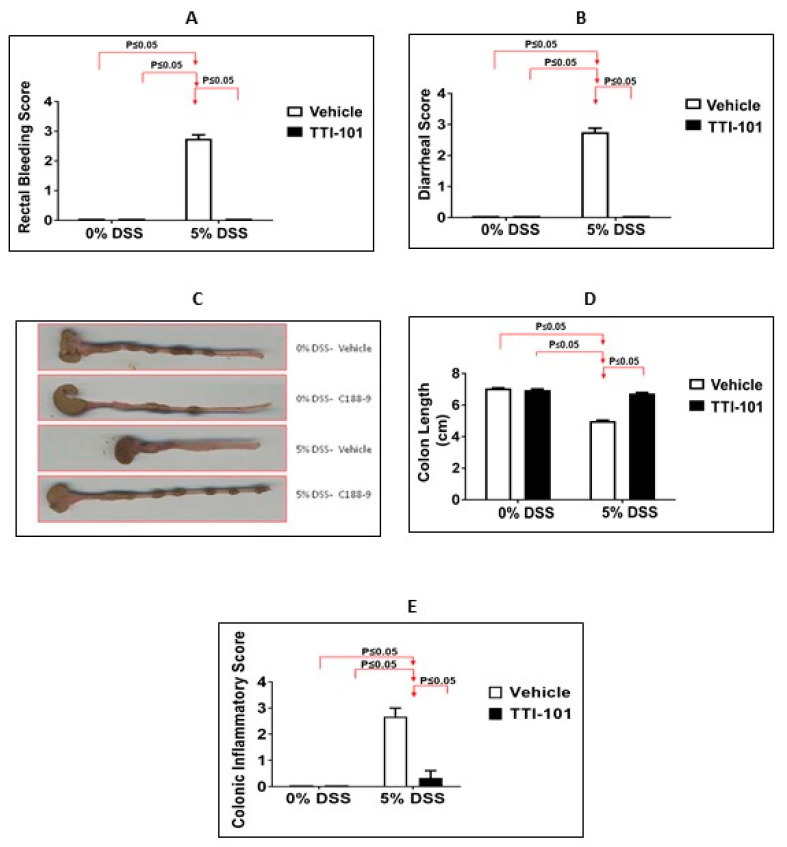
TTI-101 treatment decreases rectal bleeding, diarrhea, and colon inflammation and increases colon length in DSS-induced colitis. (**A**) Rectal-bleeding, (**B**) diarrhea, (**C**,**D**) colon shortening, and (**E**) colon inflammation scores in mice administered plain water (0% DSS) with or without TTI-101 and 5% DSS-containing water and treated with or without TTI-101. Results are expressed as the mean ± SEM of two separate experiments (all, *p* ≤ 0.05, (**A**,**B**,**E**) (Mann-Whitney test) and (**D**) (ANOVA with Tukey’s post-hoc test); n = 3–12).

The administration of TTI-101 to mice administered DSS also: (1) normalized scores for rectal bleeding and diarrhea (Figure 4A,B; *p* ≤ 0.05 for both, Mann-Whitney test; n = 9–12); (2) nearly normalized colon length (Figure 4C,D; *p* ≤ 0.05, ANOVA with Tukey’s post-hoc test; n = 9–12); and (3) reduced colonic inflammation to levels indistinguishable from controls (Figure 4E; *p* ≤ 0.05, Mann-Whitney test; n = 3–7).

**Figure 5:**
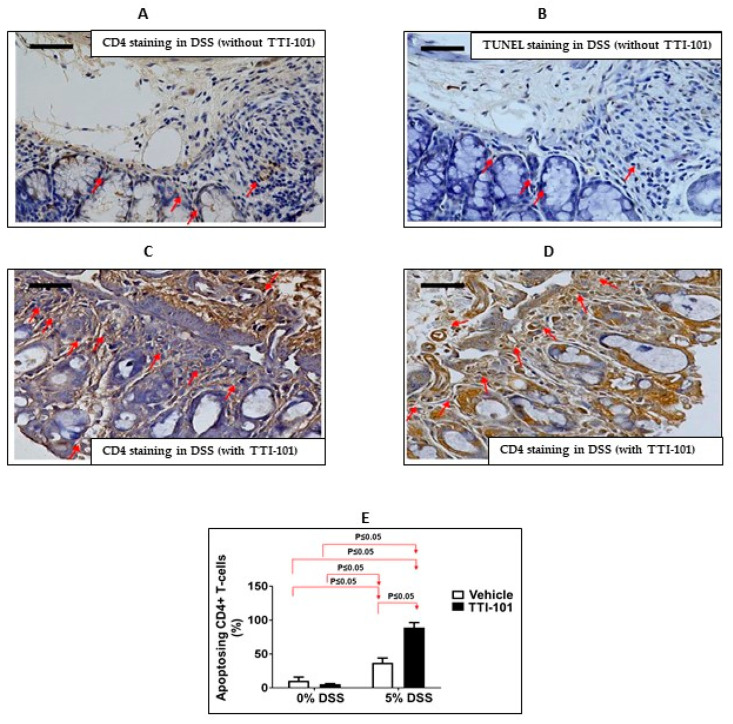
TTI-101 treatment increases the levels of CD4+ cells undergoing apoptosis in DSS-colitis. (**A**,**B**) Representative photomicrographs of CD4 (**A**) and TUNEL-stained sections (**B**) of colon tissue obtained from DSS mice without TTI-101; and (**C**,**D**) Representative photomicrographs of CD4 (**C**) and TUNEL-stained sections (**D**) of colon tissue obtained from DSS mice with TTI-101 treatment (magnification 400×; scale bar represents 50 microns). Red arrows depict cells positively stained for CD4 or TUNEL. (**E**) Percentage of apoptotic colonic CD4+ cells in mice administered plain water (0% DSS) with or without TTI-101 treatment and mice administered 5% DSS with or without TTI-101 treatment. Results are expressed as the percentage of TUNEL-positive CD4+ cells expressed as % of apoptotic CD4 T-cells ± SEM. The percentage of apoptotic CD4 T-cells was determined in ≥5 fields from n = 2–4 mice (*p* ≤ 0.05, Mann-Whitney test).

We also determined that TTI-101 treatment increased the percentage of apoptotic CD4+ T-cells in response to DSS compared to untreated mice (89.1% ± 7.4% vs. 37.1% ± 6.9%; Figure 5; *p* ≤ 0.05, Mann-Whitney test, n = 2–4).

**Figure 6:**
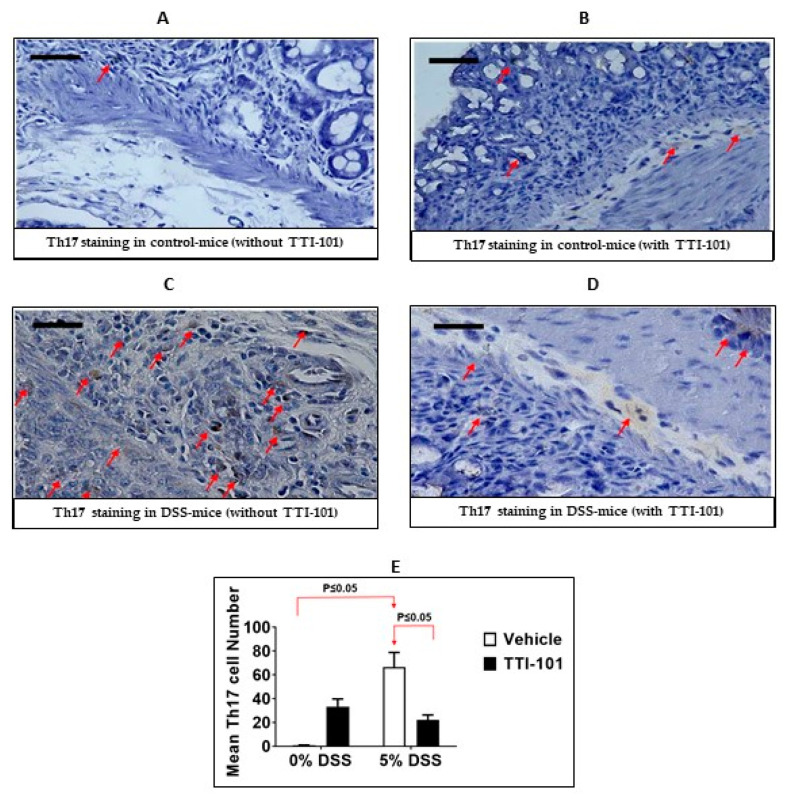
TTI-101 treatment decreases colonic IL-17-positive cell infiltration. Representative photomicrographs of sections of colons from mice administered (**A**) plain water (0% DSS) without TTI-101 treatment (**B**) plain water (0% DSS) with TTI-101 treatment; (**C**) DSS-containing water without TTI-101 treatment; and (**D**) DSS-containing water with TTI-101 treatment (magnification 400×; scale bar represents 50 microns). Red arrows depict cells positively stained for IL-17 (intracellular within the cytoplasm). (**E**) TTI-101 treatment significantly decreased levels of DSS-induced colonic IL-17-positive cell infiltration. Results are expressed as Th17 cell number ± SEM. The Th17 cell numbers are determined in ≥5 fields from n = 2–5 mice (all, *p* ≤ 0.05, Mann-Whitney test).

In addition to being implicated in colitis [7], CD4+Th17 cells have been shown to depend on STAT3 for their normal development [38]. To determine the effects of TTI-101 treatment on the number of IL-17-producing cells in DSS-colitis, we determined levels of IL-17-positive cells in DSS mice that were treated without or with TTI-101 treatment. DSS administration significantly increased the number of colonic IL-17-positive cells compared to mice given plain water (66.64 ± 12.25 vs. 0.75 ± 0.25; Figure 6; *p* ≤ 0.05, Mann-Whitney test, n = 2–5). TTI-101 treatment decreased the number of colonic IL-17-positive cells (22 ± 4) by 78% (Figure 6; *p* ≤ 0.05; Mann-Whitney test; n = 2–5).

### 3.3. Effects of TTI-101 Treatment on Colon mRNA Levels

**Figure 7:**
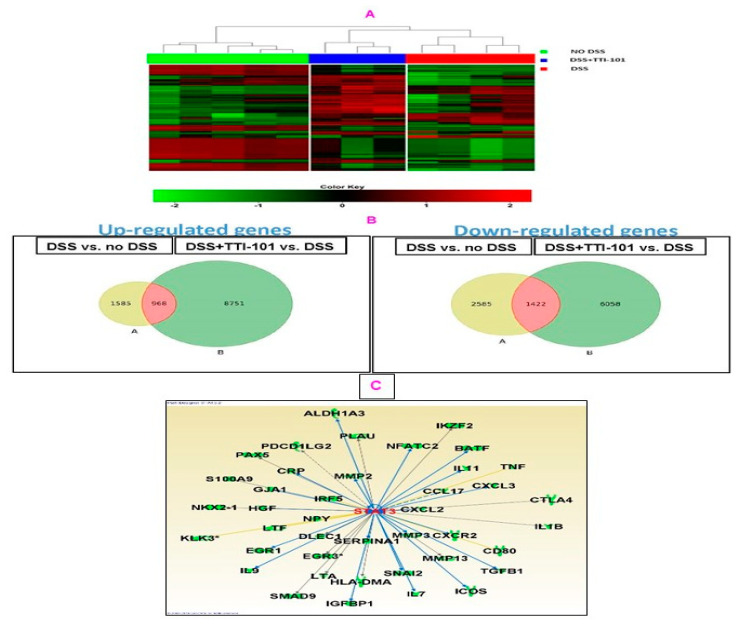
Effect of TTI-101 treatment on gene expression in DSS-induced colitis (**A**) Hierarchical clustering and heat map of differentially expressed genes in samples from three groups: no DSS, DSS, and DSS + TTI-101. (**B**) Venn diagrams show upregulated and downregulated genes in the No DSS vs. DSS and in the DSS vs. DSS + TTI-101 treatment groups. (**C**) STAT3-targeted genes determined using analysis with Ingenuity Systems’ “IPA” software; Version 4.

In order to determine the mechanisms by which TTI-101 mediates a protective effect in DSS-colitis, we performed transcriptome analyses. Hierarchical clustering of the top 500 most variable genes across all samples based on differentially expressed genes (DEGs) distinguished three main clusters corresponding to No DSS, DSS + TTI-101, and DSS (Figure 7A). The widest separation was demonstrated between the No DSS and DSS groups, followed by that between the DSS and DSS + TTI-101 groups.

To identify candidate mRNAs that contributed to DSS-induced colitis, we first performed two comparisons: DSS vs. no DSS mRNA sets and DSS + TTI-101 vs. no DSS mRNA sets and identified those genes that were differentially expressed (Figure 7B). Included in the comparison were all coding and multiple complex genes that were present in all samples of both sets used in each comparison. The criteria for differential expression were *p*-values ≤ 0.05 and FDR ≤ 0.1. We then focused on the two groups of overlapping mRNAs—those increased by DSS that were decreased by TTI-101 (968 genes; Figure 7B, middle left panel) and those decreased by DSS that were increased by TTI-101 (1422 genes; Figure 7B, middle right panel)—and performed Ingenuity Pathway Analysis (IPA) on each group. Among the 968 genes that were increased by DSS and decreased by TTI-101, 41 were STAT3 gene targets. Of the 41 STAT3 gene targets, the change in expression of 35 is consistent with the inhibition of STAT3 by TTI-101 (Figure 7C). Included among these 35 gene targets (Table 1) are: (1) 28 pro-inflammatory genes (e.g., IL-1B, CRP, CCL17, CXCL2, CXCL3, and CXCR2); (2) 12 genes previously associated with prevention of T-cell specific apoptosis (CRP, CXCL2, EGR1, ICOS, IKZF2, IL7, IL9, IL11, IL1B, NFATC2, PDCD1LG2, and TGFB); and (3) 14 genes associated with colorectal cancer metastases (CRP, CTLA4, CXCL2, CXCL3, CXCR2, GJA1, HGF, IL11, MMP2, MMP3, MMP13, S100A9, SNAI2, and TGF-β). In addition, 37 of 968 genes increased by DSS and decreased by TTI-101 (Table 2) were IBD-associated genes (e.g., CCL17, CRP, CXCL2, CXCL3, IL7, IL1β, IRF5, LTF and TNF-α). Furthermore, we determined that 1422 genes were decreased by DSS and increased by TTI-101. Among these 1422 genes, IPA analyses revealed that the expression of 7 genes decreased in response to DSS and increased with TTI-101. were associated with prevention/protection against large intestine neoplasms, including GUCA2A, CDX2, VDR, VEFGA, GSTM1, HPGD, and PPARG.

## 4. Discussion

We assessed the contribution of STAT3 to IBD by examining manifestations of DSS-induced colitis in mice genetically engineered to express only STAT3α, the more pro-inflammatory and anti-apoptotic of STAT3′s two isoforms, as well as by examining the effects of administration of TTI-101, a small-molecule inhibitor of both isoforms of STAT3. We demonstrated that all manifestations of DSS-induced colitis examined were more severe in mice expressing only STAT3α compared to wild-type mice and that TTI-101 administration completely prevented colitis through normalization of DSS-induced increases in pY-STAT3-levels within both the epithelial and stromal cell compartments. Apoptosis of colonic CD4+ T cells induced by DSS was reduced in mice expressing only STAT3α and increased in mice treated with TTI-101. TTI-101 treatment reduced the number of IL-17-positive cells infiltrating the colon and modulated levels of DSS-induced mRNA transcripts involved in inflammation, apoptosis, colorectal cancer metastasis, and large intestinal neoplasms. These results confirm other studies showing that STAT3 contributes to the pathogenesis of DSS-induced colitis, at least in part through increased survival of CD4+ cells and infiltration of IL-17-positive cells into the colon. Furthermore, these results suggest that targeting STAT3 using small-molecule inhibitors such as TTI-101 deserves consideration as a new approach to the treatment of IBD, particularly in patients refractory to current therapies.

Our results significantly expand on those obtained previously in DSS-treated Δβ/Δβ mice [24]. We report here for the first time: (1) increased DSS-induced rectal bleeding; (2) increased mortality; and (3) decreased apoptosis of colonic CD4+ T-cells in DSS-treated Δβ/Δβ mice compared to DSS-treated wild-type mice. Rectal bleeding and mortality may not have been observed in the previous study since mice were administered 2.5% DSS rather than 5% DSS, which we used in our study.

The contribution of STAT3 to IBD pathogenesis is complex as it involves distinct and opposing contributions to disease pathogenesis made by STAT3 within three different cell compartments—enterocytes, myeloid cells, and T cells. Mice in which STAT3 is conditionally inactivated in either myeloid cells or enterocytes develop enterocolitis following injections of 250 μg of dsRNA polyInosine-polyCytosine (pIpC) through triggering endogenous production of type I interferon [8]. In contrast, the inactivation of IL-6/STAT3 signaling in T cells has been shown to result in increased T-cell apoptosis accompanied by attenuation of intestinal inflammation in mouse models of IBD [9,10,11]. The results of our studies examining the impact of modulating STAT3 activity, genetically or pharmacologically, in all three cell compartments suggest a dominant role for STAT3 within the T cell compartment in the pathogenesis of DSS-induced colitis. In addition to inducing apoptosis of CD4+ T-cells, TTI-101 reduced the infiltration of IL-17-producing cells within the colon. Using a T-cell transfer model of colitis, other studies have shown that STAT3 within T cells is essential for driving colitis via the induction of Th17 cell responses [39]. Together with these findings, our results suggest that STAT3 inhibition within the T cells, specifically, perhaps, within CD4+/Th17 cells, contributes to the decreased severity of DSS-colitis in TTI-101-treated mice.

Using Affymetrix microarray technology, we identified alterations in gene expression in the colon upon TTI-101 treatment in DSS-induced colitis. TTI-101 treatment decreased the expression of 35 STAT3-associated genes whose expression was increased by DSS; of these 35 genes, 28 are associated with pro-inflammatory responses (Table 1). The 28 STAT3-regulated genes also included 12 genes associated with the prevention of apoptosis in T cells. TTI-101 treatment also led to the downregulation of 37 genes associated with inflammatory bowel disease (Table 2). These gene sets help to explain the beneficial effect of TTI-101 on colitis. Additionally, 14 of the 35 STAT3-regulated genes that were downregulated in response to TTI-101 treatment have been shown to be associated with colorectal cancer metastases [40,41,42,43,44,45,46,47,48,49,50,51,52], such as CRP, CTLA4, CXCL2, CXCL3, CXCR2, GJA1, HGF, IL11, MMP2, MMP3, MMP13, S100A9, SNAI2, and TGF-β (Table 1). Furthermore, TTI-101 treatment also led to the upregulation of important genes such as GUCA2A, CDX2, VDR, GSTM1, HPGD, and PPARG that have been shown by others to be associated with the prevention of large intestine neoplasms [53,54,55,56,57,58,59]. These last two gene sets support the possibility that STAT3 targeting in IBD may reduce the risk of CRC development. In support of the potential benefit of targeting STAT3 in diseases that predispose to CRC, there are three recent studies implicating STAT3′s contribution to CRC. Ziegler (2018), using a STAT3 loss-of-function approach in two models of intestinal tumorigenesis, demonstrated that STAT3 targeting reduced colorectal carcinogenesis by increasing mitophagy and MHC class I antigen presentation in intestinal epithelial cells, which increased anti-tumor immunity through increased activation of CD8+ T cells [60]. Studies by Poffenberger (2018) demonstrated that targeting STAT3 signaling reduced polyp growth in animals that carry germline mutations of SKT11, a gene encoding the tumor suppressor liver kinase B1 (LKB1) that is mutated in patients with Peutz-Jeghers syndrome, a disease characterized by the development of gastrointestinal polyps that predispose to CRC [61]. The third study by Schulz-Heddergott [62] demonstrated that STAT3 is activated in epithelial cells containing p53 gain of function (GOF) mutants and contributes to CRC development.

## 5. General Conclusions

In the current studies, we determined that DSS-induced colitis is more severe in mice that express unopposed STAT3α compared to wild-type mice. Importantly, we determined that a selective, small-molecule inhibitor of STAT3, TTI-101, completely prevented DSS-induced colitis as well as the upregulation of CRC-associated genes. These studies indicate that small-molecule targeting of STAT3 may be beneficial in the treatment of colitis and in the prevention of colitis-associated colorectal cancer.

## Figures and Tables

**Table 1:** STAT3 gene targets increased by DSS and decreased by TTI-101.

Symbol	Entrez Gene Name	DSS vs. No DSS	DSS + TTI-101 vs. DSS	Inflammation	T-Cell Apoptosis	Metastases
ALDH1A3	aldehyde dehydrogenase 1 family member A3	1.64	−2.12	PRO-INF	No Role	No Role
BATF	basic leucine zipper ATF-like transcription factor	1.39	−1.63	PRO-INF	No Role	No Role
CCL17	C-C motif chemokine ligand 17	1.59	−1.55	PRO-INF	No Role	No Role
CRP	C-reactive protein	1.22	−1.25	PRO-INF	ANTI-APOP	PRO-MET
CTLA4	cytotoxic T-lymphocyte associated protein 4	1.18	−1.37	ANTI-INF	PRO-APOP	PRO-MET
CXCL2	C-X-C motif chemokine ligand 2	1.91	−1.82	PRO-INF	ANTI-APOP	PRO-MET
CXCL3	C-X-C motif chemokine ligand 3	1.3	−1.47	PRO-INF	No Role	PRO-MET
CXCR2	C-X-C motif chemokine receptor 2	1.52	−1.19	PRO-INF	No Role	PRO-MET
DLEC1	deleted in lung and esophageal cancer 1	1.26	−1.45	No Role	No Role	No Role
EGR3	early growth response 3	1.4	−2.12	ANTI-INF	No Role	Anti-MET
EGR1	early growth response 1	1.57	−1.65	PRO-INF	ANTI-APOP	Anti-MET
GJA1	gap junction protein alpha 1	1.19	−1.19	PRO-INF	No Role	PRO-MET
HGF	hepatocyte growth factor	1.39	−1.35	PRO-INF	No Role	PRO-MET
HLA-DMA	major histocompatibility complex, class II, DM alpha	1.26	−1.45	No Role	No Role	No Role
ICOS	inducible T-cell costimulator	1.16	−1.28	PRO-INF	ANTI-APOP	No Role
IGFBP1	insulin-like growth factor binding protein 1	1.39	−1.41	PRO-INF	No Role	No Role
IKZF2	IKAROS family zinc finger 2	1.21	−1.27	PRO-INF	ANTI-APOP	No Role
IL7	interleukin 7	1.42	−1.22	PRO-INF	ANTI-APOP	No Role
IL9	interleukin 9	1.48	−1.41	PRO-INF	ANTI-APOP	No Role
IL11	interleukin 11	1.18	−1.42	PRO-INF	ANTI-APOP	PRO-MET
IL1B	interleukin 1 beta	1.59	−1.34	PRO-INF	ANTI-APOP	No Role
LTA	lymphotoxin alpha	1.43	−1.71	PRO-INF	No Role	Anti-MET
MMP2	matrix metallopeptidase 2	1.32	−1.24	PRO-INF	No Role	PRO-MET
MMP3	matrix metallopeptidase 3	2.48	−2.17	PRO-INF	No Role	PRO-MET
MMP13	matrix metallopeptidase 13	1.58	−1.73	PRO-INF	No Role	PRO-MET
NFATC2	nuclear factor of activated T-cells 2	1.18	−1.42	PRO-INF	ANTI-APOP	No Role
NKX2-1	NK2 homeobox 1	1.31	−1.99	ANTI-INF	No Role	No Role
PDCD1LG2	programmed cell death 1 ligand 2	1.29	−1.46	ANTI-INF	ANTI-APOP	No Role
PLAU	plasminogen activator, urokinase	1.38	−1.58	PRO-INF	No Role	No Role
PAX5	paired box 5	1.34	−2.34	No Role	No Role	No Role
S100A9	S100 calcium binding protein A9	1.58	−1.71	PRO-INF	No Role	PRO-MET
SERPINA1	serpin family A member 1	1.3	−1.47	PRO-INF	No Role	No Role
SMAD9	SMAD family member 9	1.23	−1.38	PRO/ANTI-INF	No Role	No Role
SNAI2	snail family transcriptional repressor 2	1.34	−1.12	PRO-INF	No Role	PRO-MET
TGFB1	transforming growth factor beta 1	1.22	−1.33	PRO-INF/ANTI-INF	ANTI-APOP	PRO-MET

Results are expressed as the mean SEM of two separate experiments.

**Table 2:** List of genes associated with inflammatory bowel disease.

Symbol	Entrez Gene Name	DSS vs. No DSS	DSS + TTI-101 vs. DSS
ALB	albumin	1.21	−1.31
ATP4B	ATPase H+/K+ transporting beta subunit	1.48	−2.2
BPI	bactericidal/permeability-increasing protein	1.15	−1.4
C19orf57	chromosome 19 open reading frame 57	1.38	−1.65
CCL17	C-C motif chemokine ligand 17	1.59	−1.55
CD80	CD80 molecule	1.21	−1.34
CRP	C-reactive protein	1.22	−1.25
CSF2RB	colony stimulating factor 2 receptor beta common subunit	1.85	−1.48
CXCL2	C-X-C motif chemokine ligand 2	1.91	−1.82
CXCL3	C-X-C motif chemokine ligand 3	1.3	−1.47
GABRB2	gamma-aminobutyric acid type A receptor beta2 subunit	1.24	−1.38
GABRR2	gamma-aminobutyric acid type A receptor rho2 subunit	1.28	−1.65
GABRR3	gamma-aminobutyric acid type A receptor rho3 subunit (gene/pseudogene)	1.27	−1.49
GIPR	gastric inhibitory polypeptide receptor	1.22	−1.56
GPER1	G protein-coupled estrogen receptor 1	1.38	−1.61
GRIN2D	glutamate ionotropic receptor NMDA type subunit 2D	1.35	−1.78
ICOSLG/LOC102723996	inducible T-cell costimulator ligand	1.26	−1.22
IL7	interleukin 7	1.42	−1.22
IL25	interleukin 25	1.44	−1.68
IL1B	interleukin 1 beta	1.59	−1.34
IL1RL1	interleukin 1 receptor like 1	1.24	−1.35
IL3RA	interleukin 3 receptor subunit alpha	1.43	−1.89
IRF5	interferon regulatory factor 5	1.21	−1.38
LTF	lactotransferrin	1.45	−1.59
LY6E	lymphocyte antigen 6 family member E	1.24	−1.33
MMP2	matrix metallopeptidase 2	1.32	−1.24
ORMDL3	ORMDL sphingolipid biosynthesis regulator 3	1.3	−1.69
PDCD1LG2	programmed cell death 1 ligand 2	1.29	−1.46
PPP2R2B	protein phosphatase 2 regulatory subunit Bbeta	1.17	−1.19
SLC24A4	solute carrier family 24 members 4	1.39	−1.47
SLC6A3	solute carrier family 6 members 3	1.12	−1.28
SLPI	secretory leukocyte peptidase inhibitor	1.78	−2.06
SNX20	sorting nexin 20	1.57	−2
TGFB1	transforming growth factor beta 1	1.22	−1.33
TNF	tumor necrosis factor	1.29	−1.43
TNFSF8	TNF superfamily member 8	1.42	−1.72
TREM1	triggering receptor expressed on myeloid cells 1	1.32	−1.31

Results are expressed as the mean SEM of two separate experiments.

## Data Availability

The array data has been uploaded onto the NCBI GEO database with accession number GSE117355.
